# Redox proteomic identification of carbonylated proteins in autism plasma: insight into oxidative stress and its related biomarkers in autism

**DOI:** 10.1186/s12014-017-9138-0

**Published:** 2017-01-09

**Authors:** Chengyun Feng, Youjiao Chen, Jintao Pan, Aochu Yang, Li Niu, Jie Min, Xianling Meng, Liping Liao, Kaoyuan Zhang, Liming Shen

**Affiliations:** 1 College of Life Sciences and Oceanography, Shenzhen University, Shenzhen, 518060 People’s Republic of China; 2 Early Childhood Development Center, Populations and Family Planning Hospital of Baoan, Shenzhen, 518101 People’s Republic of China

**Keywords:** Autism, Biomarkers, Plasma, Protein carbonylation, Redox proteomics

## Abstract

**Background:**

Autism is a severe childhood neurological disorder with poorly understood etiology and pathology. Currently, there is no authentic laboratory test to confirm the diagnosis of autism. Oxidative damage may play a central role in the pathogenesis of autism. Present study is an effort to search for possible biomarkers of autism and further clarify the molecular changes associated with oxidative stress that occurs in the plasma of autistic children.

**Methods:**

We performed redox proteomics analysis to compare carbonylated proteins in the plasma of autistic subjects and healthy controls. Immunoprecipitation and Western blot analysis were used to validate carbonylated proteins identified by the redox proteomics.

**Results:**

Protein carbonylation levels in two proteins, complement component C8 alpha chain and Ig kappa chain C were found to be significantly increased in autistic patients compared with controls. These two proteins were successfully validated via immunoprecipitation and Western blot analysis.

**Conclusions:**

The results further highlight the role of oxidative stress in the pathogenesis of autism and provide some information for the diagnosis and/or monitoring of autism.

## Background

Autism spectrum disorders (ASDs) are characterized by social deficits, repetitive/stereotypical behaviors and interests, as well as communication problems [1]. An increase in the prevalence of ASD is being reported worldwide with social, behavioral and economical burdens, and recent epidemiological studies indicated that at least one in every 100 people has some form of autism [2, 3]. Even more, autism related disorders are increasing at an alarming rate and have now affected 2% of US school-aged children [4]. The boys had a higher prevalence than girls and the boy to girl’s ratio on average is 4.3:1 [5].

ASD is considered as a multi-factorial disorder, influenced by genetic, neurological, environmental and immunological factors. Biomolecular evidence points to complex gene–environmental interactions in ASDs. A significant contribution from environmental factors in determining ASD risk is consistent with both the rapid increase in ASD incidence and the clinical heterogeneity which are hallmark of this neurodevelopmental disorder [6]. Several biochemical processes are associated with ASDs. These include oxidative stress, decreased methylation capacity, limited production of glutathione, mitochondrial dysfunction, intestinal dysbiosis, increased toxic metal burden and various immune abnormalities [7]. However, up to now, the etiology of autism is not fully understood, there are no medications prescribed for the core symptoms of autism. But some behavioral treatments are available to improve core and associated symptoms of autism, particularly when initiated at an early stage [8]. Regretfully, so far, no biomarkers for diagnosis or prediction of autism have been validated [9]. Thus, there is an increasing demand for finding biomarkers of autism as they could help to identify children with ASD as early as possible [10].

Human blood is a rich source for biomarker discovery. Proteomics provides the opportunity to analyze and identify biomarkers for neuropsychiatric disorders including ASD. However, in past years, very few studies are reported on proteomics based research on autism, and these were quantitative proteomics studies in which differential expression of proteins were identified [11]. Besides detecting the changes in protein concentration, post-translational protein modifications (PTMs) have contributed significantly to the identification of macromolecular biomarkers of biological processes [12]. Using a redox proteomics approach, some oxidatively modified proteins have been identified in plasma [13], cerebrospinal fluid [14] and brain of Alzheimer’s disease (AD) patients [15]. The existing evidences support the role of oxidative stress in the pathogenesis of autism [16]. Increased oxidative stress could lead to protein oxidation, resulting in 3-nitrotyrosine (3NT) and protein carbonyl formation. Protein carbonyls (PCO) and 3NT-modified proteins are considered as markers of protein oxidation. Interestingly, 3NT have been found to be elevated in plasma [17], cerebellum brains of children with ASD [18]. However, at present, no study focused on redox proteomics study of plasma proteins in autism. In order to understand the role of oxidative stress in the pathophysiology of autism and search possible protein biomarkers with diagnostic utility, we carried out redox proteomics analysis to compare protein profiles of plasma from children with autism and healthy controls in this study. To best of our knowledge, this is the first report applying redox proteomics to plasma from children with autism compared to healthy subjects.

## Methods

### Plasma samples

Twelve male and three female autistic patients (2–6 years old) were selected for sampling from Populations and Family Planning Hospital of Baoan and subjected for comparative autism analysis along with 12 male and 3 female normal control patients of the same age. The study protocol was approved by Human Research Ethics Committees of Populations and Family Planning Hospital of Baoan. The autism was diagnosed by a child neuropsychiatrist based on the criteria of autistic disorders as defined in the Diagnostic and Statistical Manual of Mental Disorder-Fourth Edition (DSM-IV). Participants did not have any physical disabilities, or additional psychiatric or neurological diagnosis or family history of ASD. They were also not taking medications and any dietary supplements. There were no significant differences in the distributions of weight, height or body mass index (BMI) between the autism and the control groups. The experiments were conducted with the written consent of the caretakers of the children under observation according to the guidelines of the Human Research Ethics Committees of Populations and Family Planning Hospital of Baoan. Blood samples (5 ml) were collected in sodium heparin coated plastic tubes in the morning and in the fasting state, and then centrifuged at 3000×*g* for 10 min at room temperature. The supernatants were divided and stored in aliquots at −80 °C for further analysis.

### Sample preparation

For redox proteomic analysis, the pooled plasma samples were used. Equal amounts of plasma from 15 autism and 15 healthy controls were pooled, respectively. In order to reduce the sample complexity, the pooled plasma samples were pre-treated with ProteoExtract Albumin/IgG Removal Kit (Calbiochem, Darmstadt, Germany). After high-abundance proteins depletion, the samples were centrifuged at 12,000×*g* in Amicon^®^ Ultra Centrifugal Filters (3 kDa cut-off, Millipore) and buffer-exchanged with sample buffer (7 M urea, 2 M thiourea, 4% (w/v) CHAPS, 2% (v/v) immobilized pH gradients (IPG) buffer pH 4–7 NL, 65 mM DTT, 30 mM Tris). To verify the efficiency of immunodepletion, flow-through fraction (depleted serum) and eluted fraction were separated on sodium dodecyl sulfate–polyacrylamide gel electrophoresis (SDS–PAGE) followed by Coomassie brilliantlue staining R-250. All protein samples were stored at −80 °C until further analysis and the protein concentration was determined by the Bradford assay.

### 2D-Oxyblot

PCO were analyzed by 2-DE (two-dimensional gel electrophoresis) plus Western blot analysis (2D-Oxyblot) using the in-strip derivatization technique [19, 20]. 2-DE was performed as described previously in detail [21]. Each sample was electrophorized in duplicates, after running, proteins in one gel were silver stained, and in another gel was transferred to polyvinylidene difluoride (PVDF) membrane. The membranes were subsequently blocked, washed and incubated overnight at 4 °C for immunoblotting with anti-DNP (dinitrophenylhydrazone) antibody (1:1000 dilution, Sigma-Aldrich Co., St. Louis, USA). The blots were then washed with PBS (phosphate-buffered saline) and 0.2% (v/v) Tween-20 (PBST) and incubated with the goat anti-mouse IgG/HRP conjugate (1:5000 dilution, Abmart Inc, Shanghai, China). After three washes with PBST, the signal was detected with an ECL kit (Pierce ECL detection kit, Thermo Fisher Scientific Inc., Rockford, USA). The emitted chemiluminescent signals were detected using a digital imaging system (Kodak Image Station 4000MM, Carestream Health, Inc., Rochester, NY, USA). Silver-stained gels were imaged using the proXPRESS 2D imaging system (PerkinElmer, Waltham, MA, USA). The intensity of carbonylated spots on 2D-Oxyblots was normalized versus their respective spots visualized on silver stained gels. The spots showing significant differences in specific carbonylation levels between autism and control using Student’s *t* test statistical analysis (*p* < 0.05) were chosen for identification. For protein identification, the spots of interest were excised manually from the silver-stained gels and tryptic in-gel digestion was performed as described previously [22]. Mass spectroscopy analysis was performed on a 5800 MALDI TOF/TOF mass spectrometer (AB Sciex, Foster City, CA, USA) [21, 22] or Triple TOF 5600 system (AB Sciex) [23].

### Immunoprecipitation and post-Western blot derivatization

To confirm the redox proteomic results, the carbonyl levels of C8A and IGKC were detected by post-Western blot derivatization after immunoprecipitation [20].The experiment was replicated three times. For each time, three age-and sex-matched different subjects were randomly chosen from the total 15 of each group, and their original individual plasma frozen aliquots were used. C8A and IGKC were immunoprecipitated using C8A antibody (Santa Cruz Biotechnology Inc., Santa Cruz, CA, USA) and IGKC antibody (Bioss Inc., Beijing, China), respectively, and then probed for protein carbonyl levels. Protein samples (300 µg) were incubated overnight at 4 °C with the respective antibodies. Protein A and G plus-agarose beads (Santa Cruz Biotechnology Inc.) were added, and the mixture was incubated for 3 h, and then washed with lysis buffer three times. The beads were resuspended in SDS loading buffer and boiled for 5 min. After centrifugation, the supernatant was collected, separated by SDS–PAGE, and transferred to PVDF membranes. The membranes were equilibrated in solution A (20% (v/v) methanol: 80% (v/v) PBST) for 5 min, followed by incubation in 2 N HCl for 5 min. The proteins on blots were then derivatized in solution B (0.5 mM DNPH (2,4-dinitrophenylhydrazine) in 2 N HCl) for 5 min [24]. The membranes were washed three times in 2 N HCl for 5 min each and then five times with 50% methanol and two times with PBST each for 5 min. The immune complexes were revealed by enhanced chemiluminescence as described above. The data are presented as mean ± standard error of the mean (SEM) and statistical analyses were performed by two-tailed Student’s *t* test. *p* values <0.05 were considered statistically significant.

## Results

### Immunodepletion of high abundance plasma proteins

**Fig. 1:**
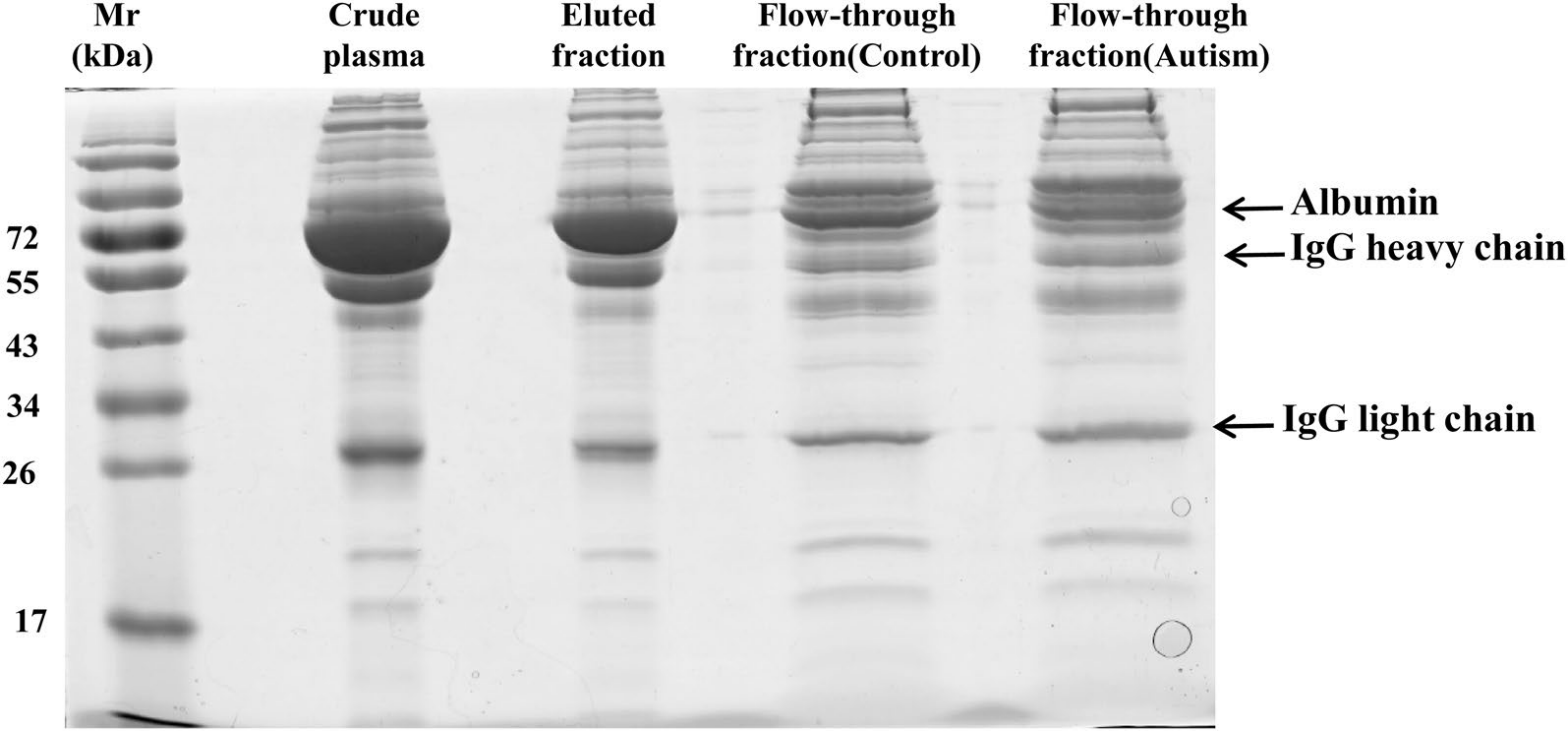
The efficiency of immunodepletion of high-abundance plasma proteins. 20 µg crude plasma (without depletion), flow-through fractions (low and medium abundance proteins) and eluted fractions (high abundance proteins) were separated on 12% SDS–PAGE gel and stained with silver. *Mr* molecular weight

The most challenging obstacle to develop blood-based biomarkers is the massive dynamic range of proteins in blood, spanning up to 12 orders of magnitude [25]. In this study, prior to proteomic analysis, plasma samples were processed using the ProteoExtract Albumin/IgG Removal Kit, which selectively removes albumin and immunoglobulin (IgG) from the plasma sample. The protein patterns of plasma samples before and after depletion were visualized on SDS–PAGE gels and shown in Fig. 1. Consistent with the user manual, after depletion of high abundance proteins as compared to the crude plasma sample, more protein bands were observed in the lane of depleted plasma, suggesting that the low and medium abundance proteins could be enriched by affinity depletion of abundant proteins. However, the results also showed that most of albumin and IgG were removed except few of them (Fig. 1), which is consistent with manufacturer’s instruction and previous study [26].

### Redox proteomics analysis of children with autism and healthy control plasma samples

**Fig. 2:**
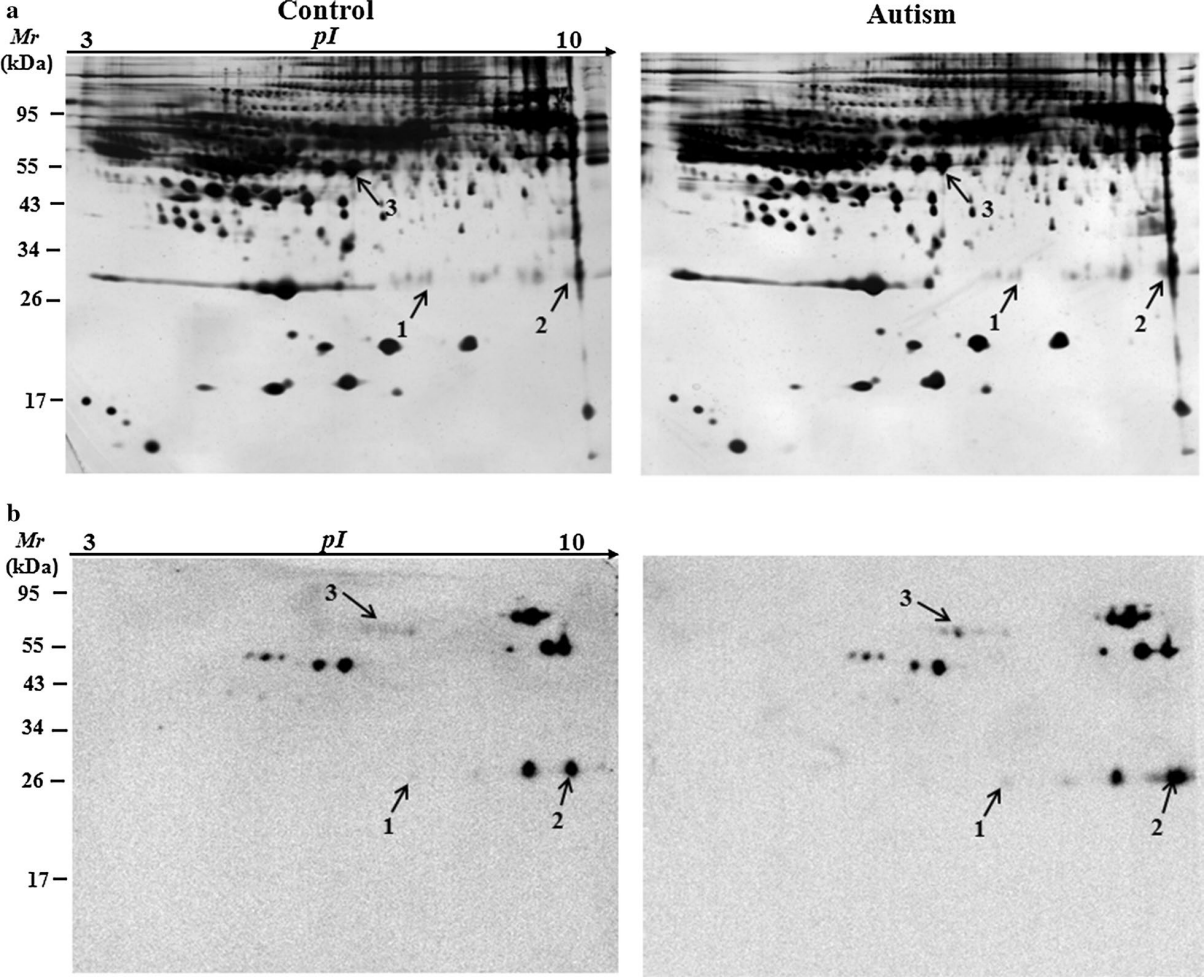
Analysis of carbonylated plasma proteins from the plasma of children with autism and age-matched control subjects by 2D-Oxyblots. **a** Representative 2-DE gel stained with silver to visualize all protein of plasma from the control and the children with autism. **b** Representative 2D-Oxyblots of plasma from the control and the children with autism

Using a redox proteomics approach, after isoelectric focusing (IEF), IPG strips were derivatized with DNPH, which were then separated by SDS–PAGE gel and detected by Western blot analysis with anti-DNP antibody. Hence the carbonylated proteins were identified. Figure 2 shows representative 2D gels with silver staining of total proteins and the corresponding 2D-Oxyblots for carbonylated proteins in the plasma of autistic children and controls. Through image comparison, three proteins were found to be significantly elevated in carbonyl levels (*p* < 0.05) and considered as carbonylated proteins in children with autism (Fig. 2b). These were identified by mass spectrometry analysis. These proteins were complement component C8 alpha chain (C8A) and Ig kappa chain C (IGKC). The relevant information of these proteins is listed in Table 1 and indicated by arrows and numbers in Fig. 2. The spots 1 and 2 were identified as the same protein (Table 1; Fig. 2), i.e., IGKC. No significant differences were observed in their protein expression levels between patients and the healthy controls (Fig. 2a).

**Table 1:** Oxidatively modified proteins identified in the plasma of children with autism compared to the age matched controls

Spot	Protein identified	Gene name	SwissProt accession	MW(kDa)/pI	Protein Score	Peptides matched^a^	Oxidation fold^b^
1	Ig kappa chain C	IGKC	P01834	11.8/5.58	2332	61 (54)	2.45
2	Ig kappa chain C	IGKC	P01834	11.8/5.58	184	3 (3)	2.06
3	Complement component C8 alpha chain	C8A	P07357	66.8/6.07	1317	43 (32)	2.18

^a^Peptides matched by mass fingerprinting

^b^ *p* < 0.05 versus the control

### Validation of redox proteomics results

**Fig. 3:**
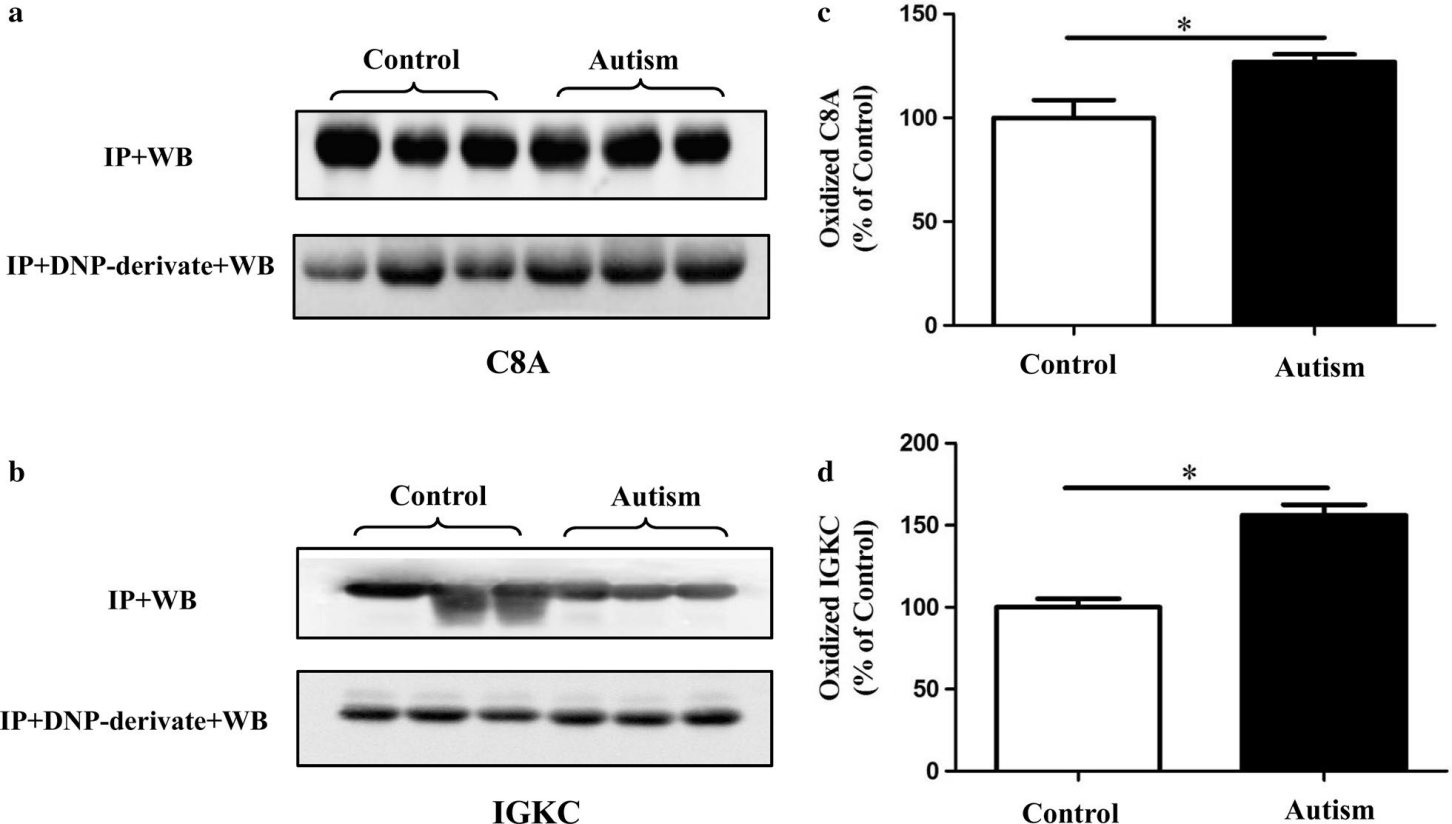
Immunoprecipitation followed by Western blot analysis was performed to confirm the carbonylation of C8A and IGKC proteins in plasma. **a**, **b** The efficiency of immunoprecipitation was checked with anti-C8A and anti-IGKC antibody (IP + WB), respectively. They were immunoprecipitated with respective antibodies and Western blot analysis with anti-DNP antibody (IP + DNP-derivate + WB). **c**, **d** Histograms represent the alteration of protein carbonyl levels in which the measured value is normalized with the mean of the control subjects. *p < 0.05 versus control

To validate redox proteomics results, the carbonyl levels of C8A and IGKC were detected. Samples were post-derivatized with DNPH on a membrane and probed with anti-DNPH antibody to identify the carbonylated proteins. Consistent with redox proteomics results, the carbonyl levels of C8A and IGKC were significantly higher in the plasma of children with autism compared with the healthy control subjects (Fig. 3, *p* < 0.05).

## Discussion

Autism is a severe developmental disorder with poorly understood etiology. Oxidative stress is documented and independently confirmed to be increased in children with autism by various methods [27]. Increased excretion of oxidative stress biomarkers and reduced levels of antioxidants have been reported in autism [16, 27–29]. The brain is highly vulnerable to oxidative stress due to its limited antioxidant capacity, higher energy requirement, and higher amounts of lipids and iron [30]. Children are more vulnerable than adults to oxidative stress because of their naturally low glutathione levels from conception through infancy [27, 31]. Oxidative stress could result in protein oxidation, however, the relation between carbonylated proteins and autism has not been investigated yet.

Here, we applied redox proteomics approaches to analyze the carbonylated proteins in the plasma of autistic patients. The results revealed that the carbonyl levels of two proteins (i.e., C8A and IGKC) were significantly increased in autistic subjects compared with age-matched controls. C8A is involved in complement and coagulation cascades and IGKC involved in immune response. Interestingly, complement active and immune dysfunction has been related to the pathogenesis of autism [32, 33]. The complement system comprises a group of proteins which, when activated, provide one of the first lines of defense by promoting lysis and removal of invading microbes [34]. The complement system may also be involved in cellular apoptosis in brain and peripheral differences of immune molecules that could impact indirectly on the developing brain in autism [32]. Comparison with healthy controls, several up-regulated complement proteins have been reported in the serum of ASD [32], including complement factor H related protein (FHR1), complement C1q and complement factor I (CFI). Increases in three peptides that correspond to C3 complement protein fragments were identified in the plasma of children with ASD [33]. Here, C8A showing significantly increased carbonyl levels in the plasma from autistic children compared with controls, demonstrating and supporting the option that complement system may be involved in the pathophysiology of autism [32, 33]. Generally, oxidative modification of proteins/enzyme leads to dysfunction or decreased activity, but the effect of oxidatively modified C8A is not clear and require further study.

Immune dysfunction such as immune cell dysfunction, imbalance of serum IgGs and cytokines has been proposed as a potential mechanism for ASD [35–37]. Decreased levels of IgM and IgG classes of IgG have been observed in a previous study, with lower levels found to correlate with more aberrant behaviors in ASD [36]. Studies showed that higher frequency of autoimmune disorders, such as rheumatoid arthritis in families with autistic probands than in those of healthy control subjects [27]. In this study, two protein spots were identified as IGKC. As the plasma used were obtained from 2 to 6 years old children, implying that IgG is prone to be oxidized. This is consistent with previous studies showing that IgG and especially its Fc portion was quite vulnerable to reactive oxygen species [38]. Elevated levels of oxidized IgG have been observed in various diseases, including AD [14], rheumatoid arthritis [39], end-stage renal disease patients [40] and type 1 diabetes mellitus etc. [41, 42]. Very recently, five IgG proteins including Ig gamma-2B chain C region (IGH-3), Ig lambda-2 chain C region (IGLC2), IGKC and Ig kappa chain V-V region HP R16.7 were identified as carbonylated proteins in the serum of 3-month-old triple transgenic AD mice (3× Tg-AD mice) [43].

IgG is the high abundant plasma protein in the plasma, which was identified as oxidatively modified protein. This shows that a relative amount of IgG was still present in the IgG-depleted plasma samples [26]. Oxidative stress alters its structure and may result in modification of its biological properties. In vitro study has demonstrated that oxidation of IgG impairs its ability to bind to macrophage Fc receptors [44] and may lead to decrease its anti-inflammatory properties. Likewise, a previous study proposed that conformational changes in IgG due to oxidative stress could render it immunogenic, resulting in induction of autoantibodies in type 1 diabetes patients [42]. Thus, we speculate that oxidative stress lead to protein oxidation modifications being one of the factors inducting autoimmune response in autistic patients.

It is necessary to point out that this study is a preliminary investigation. As oxidative stress has been related to various diseases, and thus may decrease the specificity of carbonylated protein as disease markers. However, based on the complexity of autism pathogenesis, a combination of multiple markers could be a more powerful approach to diagnose this disease [16]. Even more, analyses of both protein expression level and oxidative modification (carbonylation) could increase specificity of the marker [13]. Thus, the oxidatively modified proteins may also be considered as one type of biomarkers in blood for autism diagnosis, and/or combined with differentially expressed proteins between autistic patients and healthy subjects. In addition, the results would have been enhanced had there been another non-ASD control group with developmental delays and/or other disorders of childhood. Clearly, this needs to further investigate. Moreover, this technology and technique may also assist in monitoring disease states like autism and responses to treatment in a clinical trial environment.

## Conclusions

This is the first study by using 2D-Oxyblot analysis to investigate oxidatively modified plasma proteins in autistic children compared to healthy controls. The results showed that the carbonyl levels of two proteins (C8A and IGKC) were significantly higher in the plasma of autistic children than in healthy controls, which are found to be involved in complement system and immunoregulation. The results support the view that oxidative stress may be involved in the pathogenesis of autism and add additional evidence for oxidative stress in autism, implicating that antioxidant therapy may be beneficial in the treatment of autism. This study will enhance our understanding about autism pathogenesis, and if this can be replicated in larger independent and controlled trials then maybe this type of technique can be used in the future as a possible diagnostic tool.

## Abbreviations

2-DE: two-dimensional gel electrophoresis
3NT: 3-nitrotyrosine
AD: Alzheimer’s disease
ASD: autism spectrum disorder
DNPH: 2,4-dinitrophenylhydrazine
IPG: immobilized pH gradients
